# Simultaneous determination of myricetrin, quercitrin and afzelin in leaves of *Cercis chinensis* by a fast and effective method of ionic liquid microextraction coupled with HPLC

**DOI:** 10.1186/s13065-018-0391-8

**Published:** 2018-03-01

**Authors:** Mengjun Shi, Nan He, Wenjing Li, Changqin Li, Wenyi Kang

**Affiliations:** 1 Institute of Chinese Materia Medica, Henan University, Kaifeng, 475004 Henan China; 2 Kaifeng Key Laboratory of Functional Components in Health Food, Henan University, Kaifeng, 475004 Henan China

## Abstract

In this study, the contents of myricetrin, quercitrin and afzelin in *Cercis chinensis* leaves were determined simultaneously by 1-butyl-3-methylimidazolium tetrafluoroborate [BMIM] BF_4_/70% ethanol microextraction combined with High Performance Liquid Chromatograph (HPLC) analysis. The mobile phase was eluted with an Agilent ZORBAX SB-C18 column (4.6 mm×5 mm, 5 μm), B was methanol and C was 0.1% glacial acetic acid–water as the mobile phase. The flow rate was 0.8 mL min^−1^, eluents was detected at 245 nm at column temperature of 30 °C. The orthogonal experiment and variance analysis were used to determine the optimum process of *C. chinensis* leaves by the comprehensive evaluation of the contents of myricetrin, quercitrin and afzelin. The results showed that the injection rates of myricetrin, quercitrin and afzelin were in the range of 0.4997–18.73 μg (*r* = 0.9997), 0.1392–5.218 μg (*r* = 0.9998) and 0.04582–1.718 μg (*r* = 0.9998), respectively. The optimum conditions were determined as follows: the concentration of extraction, 0.9 mol/L; the ultrasonic time, 50 min; the solid–liquid ratio, 1:30; the centrifugal speed, 5000 r/min, and the crushing ratio, 90 mesh. Under these optimal conditions, the average levels of myricetrin, quercitrin and afzelin were 8.6915, 1.5865 and 1.0920 (mg/g), respectively.

## Introduction

*Cercis chinensis* (*C. chinensis*) belongs to family Leguminosae and is one of Chinese Materia Medica. Its root, bark, flower and fruit have pharmacological activities [1]. Its main chemical constituents were reported to be flavonoids, stilbenes, phenolic acids, lignans and cyanogenic glycosides [2–4]. Zhang et al. [5] had found that the bark of *C. chinensis* had obvious analgesic and anti-inflammatory effects. Na et al. [6] reported that the alcoholic extracts of leaves and stems of *C. chinensis* could scavenge 1,1-Diphenyl-2-picrylhydrazyl (DPPH) free radicals and inhibit lipid peroxidation induced by Fe^2+^. A total of 20 compounds were isolated by bioassay-guided method. Among them, myricetrin, quercitrin and other flavonoids had antioxidant, antitumor, hepatoprotective and other activity [7, 8].

As an effective component in medicinal plants, effective extraction of the active ingredients has been widely reported. There are many methods reported in the literature [9–12]. However, the traditional methods of organic solvent extraction are time-consuming and inefficient and cause pollution to the environment and do not complete extraction. Currently, ionic liquids (ILs), also known as room temperature molten salts, is one kind of green solvent models, which is consisted of a specific, relatively large, asymmetric organic cation and a relatively small amount of inorganic anion [13]. ILs exhibit a large number of good characteristics, including thermal stability and chemical stability, wide viscosity range, and adjustable solubility [14]. With the principle of dissolution plant cell wall, ILs could extract compounds more completely and shorten the extraction time [15–18]. Therefore, the effective extraction can be obtained by using the appropriate ILs.

**Fig. 1:**
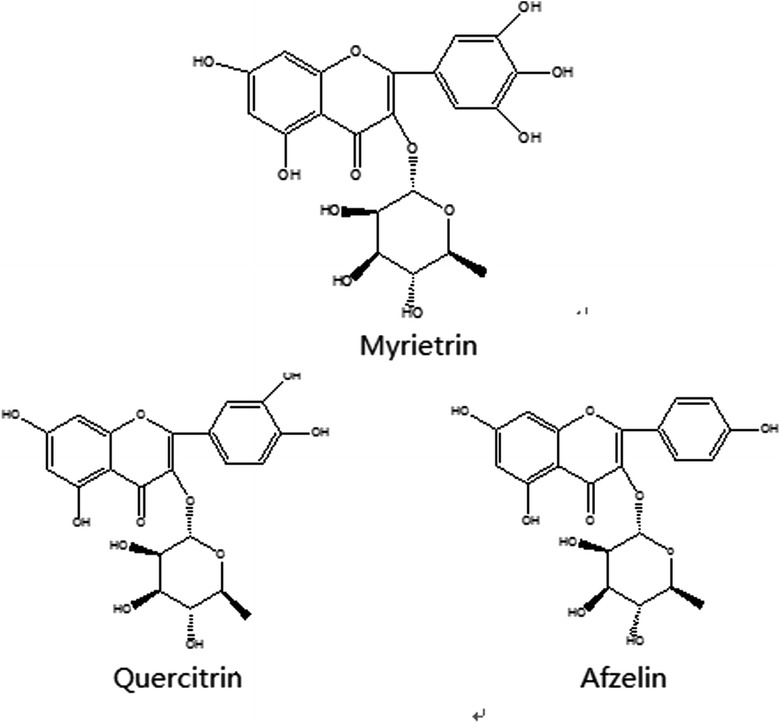
Chemical structures of myricetrin, quercitrin and afzelin

To the best of our knowledge, myricetrin, quercitrin and afzelin (Fig. 1) are the effective components in leaves of *C. chinensis*. However, there have been no reports about the ILs extraction of flavonoids, such as myricetrin and quercitrin, from leaves of *C. chinensis*. Therefore, our study aimed to establish a rapid and effective ionic liquid-based, ultrasonic-assisted extraction method (IL-UAE) combined with high performance liquid chromatography (HPLC) to separate and determine simultaneously myricetrin, quercitrin and afzelin, design orthogonal test by SPSS 19.0, screen the optimal extraction method, and carry out the investigation of methodology.

## Experimental methods

### Chemicals and materials

Methanol (chromatographic grade) was purchased from Tianjin Da Mao Chemical Reagent Factory (Tianjin, China). The ultra pure water was purchased from Hangzhou Wahaha Baili Food Co. Ltd, (Zhejiang, China). Acetic acid was obtained from Tianjin Fu Chen Chemical Reagent Factory (Tianjin, China). 1-butyl-3-methylimidazolium tetrafluoroborate ([BMIM]BF_4_), 1-butyl-3-methylimidazole bromide ([BMIM]Br) and 1-butyl-3-methylimidazolium hexafluorophosphate ([BMIM]PF_6_) were obtained from limited partnership Merck (Darmstadt, German). 1-hexyl-3-methylimidazolium hexafluorophosphate ([HMIM]PF_6_) was purchased from Termo Fisher Scientific (Rockville, MD, USA). Quercitrin with purity greater than 98% was purchased from Chengdu Pufei De Biotech Co., Ltd. Myricetrin and afzelin with purity greater than 98% were isolated in our previous chemical research.

A LC-20AT high performance liquid chromatography system (Shimadzu, Kyoto, Japan) equipped with a degasser, a quaternary gradient low pressure pump, the CTO-20A column oven, a SPD-M20AUV-detector, a SIL-20A auto sampler was used. Chromatographic separations of target analytes were performed on an Agilent ZORBAX SB-C18 column (4.6 mm×5 mm, 5 μm) and KQ-500DB ultrasonic cleaner (Jiangsu Kunshan Ultrasonic Instrument Co., Ltd. Jiangsu, China). TGL-16 type high speed centrifuge was obtained from Jiangsu Jintan Zhongda instrument factory (Jiangsu, China). AB135-S 1/10 million electronic balance was purchased from Mettler Toledo Instruments Co., Ltd (Shanghai, China).

### Plant materials and sample preparation

The leaves of *C. chinensi*s were collected in July 2016 from the campus of Henan University (Kaifeng, Henan, China) and identified by Professor Changqin Li. A voucher specimen was deposited in the Institute of Traditional Chinese Medicine, Henan University.

### Preparation of the standard solution

Three standard solutions of myricetrin, quercitrin and afzelin were prepared in methanol at a concentration of 249.86, 69.85 and 22.91 μg mL^−1^, respectively and stored at 4 °C.

### Preparation of test sample solution

The powder of *C. chinensi*s leaves (1 g, 90 mesh) was dissolved in [BMIM] BF_4_/70% ethanol (30 mL) solution using volumetric flask. The sample was extracted by ultrasonic extraction for 50 min, then centrifuged at 5000 r min^−1^ for 5 min. The supernatant was passed through a 0.22 μm organic microporous membrane. The filtrate was obtained and used as the sample solution. The type of ILs, the concentration of selected IL, the mesh sieve through which of *C. chinensi*s was passed, the ultrasonic time and solid–liquid ratio were systematically investigated in this experiment.

### Chromatographic conditions

Chromatographic conditions were set as follows: separation column, Agilent ZORBAX SB-C18 column (4.6 mm × 250 mm, 5 μm); mobile phase, methanol (B)-0.1% aceticacid (C); gradient elution (0–8 min, 35–50%B, 65–50%C; 8–25 min, 50–52%B, 50–48%C; 25–30 min, 52–55%B, 48–45%C; 30–35 min, 55–65%B, 45–35%C); column temperature, 30 °C; flow rate, 0.8 mL/min; the UV detection wavelength, 254 nm; and sample volume, 10 μL.

**Fig. 2:**
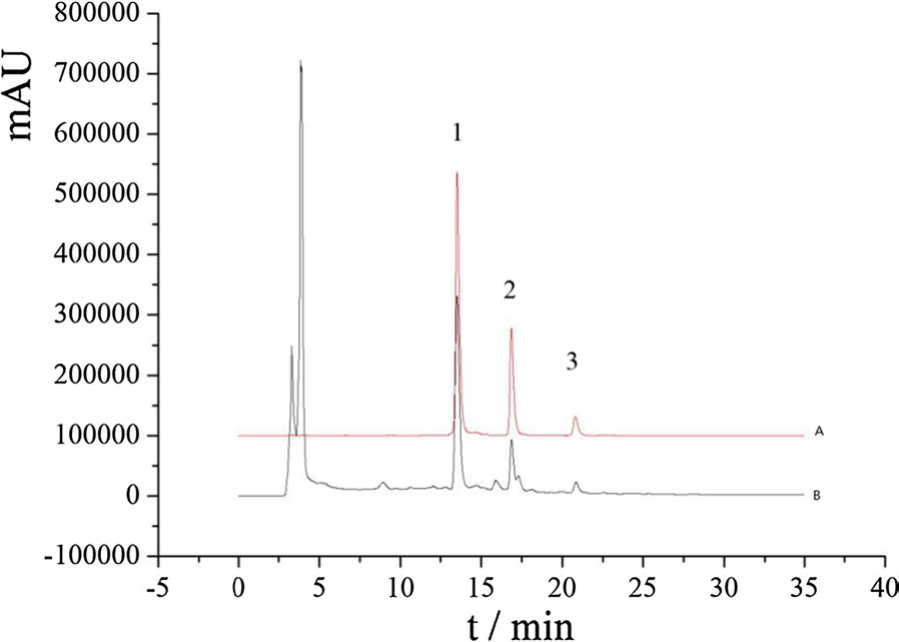
HPLC chromatograms of the standard solution (**a**) and the test sample solution (**b**):1. Myricetrin, 2. Quercitrin, 3. Afzelin

The HPLC chromatograms of the standard solution and the sample extract were shown in Fig. 2.

### Optimization extraction process of flavonoids in *C. chinensi*s leaves

The orthogonal experiments of 5 factors and 3 levels were designed by SPSS 19.0 to screen out the optimal extraction conditions of flavonoids such as myricetrin in leaves of *C. chinensi*s. In Table 1, the range of each factor level was set based on the results of preliminary experiments. The yields (%) of myricetrin, quercitrin and afzelin were taken as the dependent variables. The extraction yields of target analytes were determined with the following formula (1).

1 $$ {\text{yield}}\, ( {\text{mg/g) = }}\frac{{{\text{mean}}{\kern 1pt} \;{\text{mass}}\;{\text{of}}\;{\text{target}}\;{\text{analytes}}\;{\text{in}}\;{\text{herb}}\;{\text{samples}}\, ( {\text{mg)}}}}{{{\text{mean}}\;{\text{mass}}\;{\text{of}}\;{\text{the}}\;{\text{herb}}\;{\text{samples}}\, ( {\text{g)}}}}. $$

**Table 1:** Orthogonal test factors and level tables

Factor	A	B	C	D	E
Level	Solid–liquid ratio (Times)	Extractant concentration (mol/L)	Ultrasound time (min)	Centrifugal speed (r/min)	Crush mesh (Mesh)
1	1:20	0.5	20	3000	50
2	1:30	0.7	35	5000	70
3	1:50	0.9	50	6000	90

## Results and discussion

### Linear relationship

For preparing standard sample solutions, various amounts of myricetrin, quercitrin and afzelin were dissolved in methanol to yield their stock solutions, respectively. Corresponding calibration curves for myricetrin, quercitrin and afzelin were Y = 2896540X − 93968, (*r* = 0.9997), Y = 4208940X − 60256, (*r* = 0.9998) and Y = 2741410X − 1610.5, (*r* = 0.9999), respectively. Myricetrin, quercitrin and afzelin showed good linearity in the ranges of 0.4997–18.73 (μg/mL), 0.1392–5.218 (μg/mL) and 0.04582–1.718 (μg/mL), respectively. The limit of detection (LODs, based on signal-to-noise ratio of 3, S/N = 3) and the limit of quantifcation (LOQs, based on signal-to-noise ratio of 10, S/N = 10) of myricetrin were 13.86 and 23.55 ng, respectively; LOD and LOQ of quercitrin were 2.505 and 5.009 ng, respectively; and LOD and LOQ of afzelin were 1.099 and 2.190 ng, respectively.

### Selection period of ILs

The ILs type has a great effect on the extraction rate of target compounds. In our study, four kinds of ILs, including [BMIM]BF_4_, [BMIM]Br, [BMIM]PF_6_, and [HMIM]PF_6_, were tested as the extraction solvents. The four kinds of ILs belonging to imidazole are stable both in air and solution and can be combined with lignocellulose by competion, which could improve the efficient cellulose dissolved so that increase the rate of extraction [19]. However, ILs are mostly viscous liquids while [BMIM] Br is crystalline solid. Thus, it is important to select suitable solvents to dissolve ILs.

**Fig. 3:**
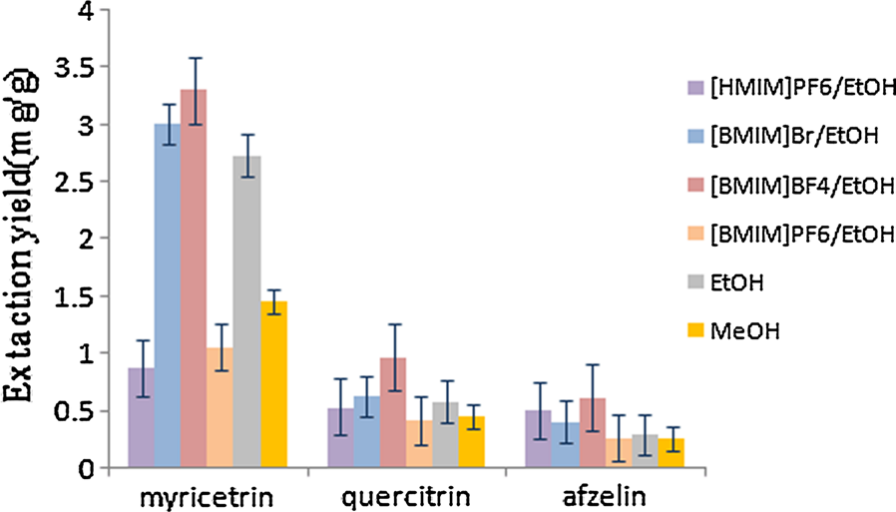
Effect of extraction solvents (n = 3)

70% Ethanol (EtOH), methanol (MeOH), acetonitrile and water were compared. Each experiment was paralleled three times. The results showed that water and acetonitrile were not suitable to be used to extract flavonoids from the leaves of *C. chinensi*s. Because the myricetrin, quercitrin and afzelin in acetonitrile and water extract did not appear in the HPLC. In Fig. 3, EtOH was the best solvent for extracting target analytes. Therefore, 70% EtOH was selected as the solvent in the following studies.

The effects of four kind of ILs with EtOH on target analytes were compared and the results are displayed in Fig. 3. It showed that the highest extraction rate of target analytes was obtained by using [BMIM]BF_4_/EtOH, which may be related to the composition and structure of ionic liquids.

### Effect of concentrations of the ILs selected

**Fig. 4:**
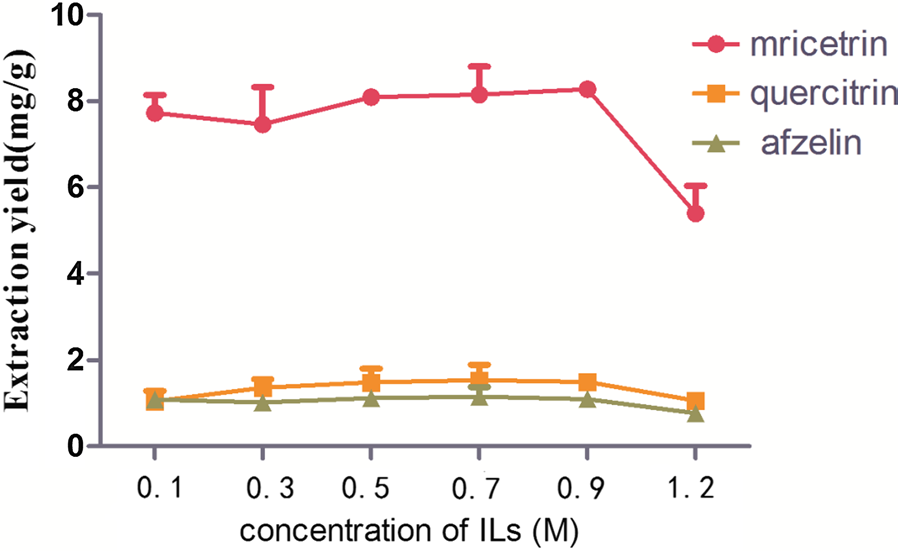
Effect of concentration of ILs (n = 3)

In Fig. 4, there was a positive correlation between the extraction yields of the target compounds and the IL concentration ranged from 0.1 to 0.7 M. But over 0.7 M, the more ILS was used, the fewer target compounds were obtained. It indicated that the diffusion force of the solvent was decreased when the concentration of ionic liquids was increased, and it was hard to enter the internal, and the ingredients could not be fully extracted from the medicinal herbs. Thus, extraction rate was decreased [20, 21]. Results suggested that 0.7 M was chosen as the optimum IL concentration. Each experiment was paralleled three times.

### Selection of particle size

**Fig. 5:**
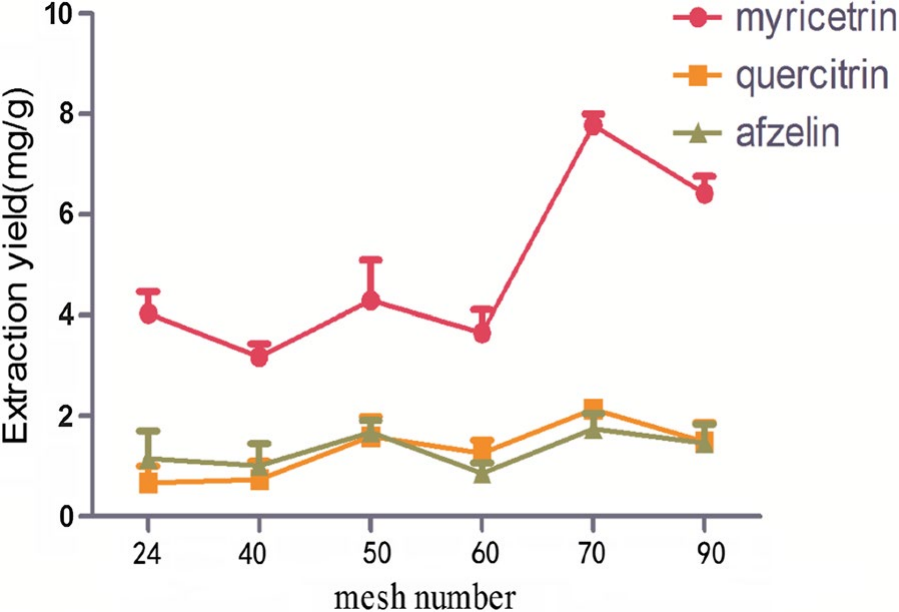
Effect of mesh numbers on extraction yield (n = 3)

The leaf powder of *C. chinensis,* passed through 24, 40, 50, 60 70 and 90 mesh, was investigated. Each experiment was paralleled three times. In Fig. 5, with the increase of grinding mesh, extraction yields from leaf powder of *C. chinensis* were increased till the myricetrin, quercitrin and afzelin extraction rate reached the maximum at 70 mess. The results indicated that the leaf constituent of *C. chinensis* is easy to be extracted with the decrease of viscosity, but if the particle size is too small, it would hinder the release of its chemical constituents by the ionic liquid quality [19].

### Effect of ultrasonic time

**Fig. 6:**
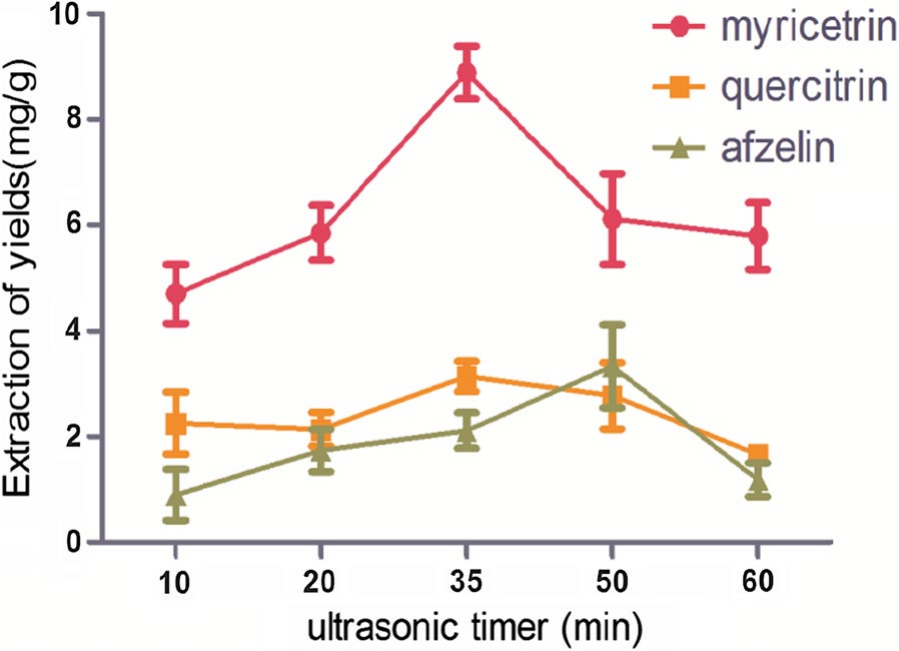
Effect of ultrasonic times on extraction yield (n = 3)

In Fig. 6, with the extension of ultrasonic extraction time, extraction rate of target compounds was increased gradually. Each experiment was paralleled three times. The extraction yields of myricetrin, quercitrin and afzelin in leaves of *C. chinensi*s reached the maximum at 35 min. Then, as time increased, the extraction rates of three target compounds were decreased. This may be due to the reason that prolonging ultrasonic extraction time will destroy the structure of ILs and target analytes [22], but the specific and exact reasons need to be further studied. Thus, the ultrasonic time for 35 min was chosen as the optimal condition.

### Effect of solid–liquid ratio

**Fig. 7:**
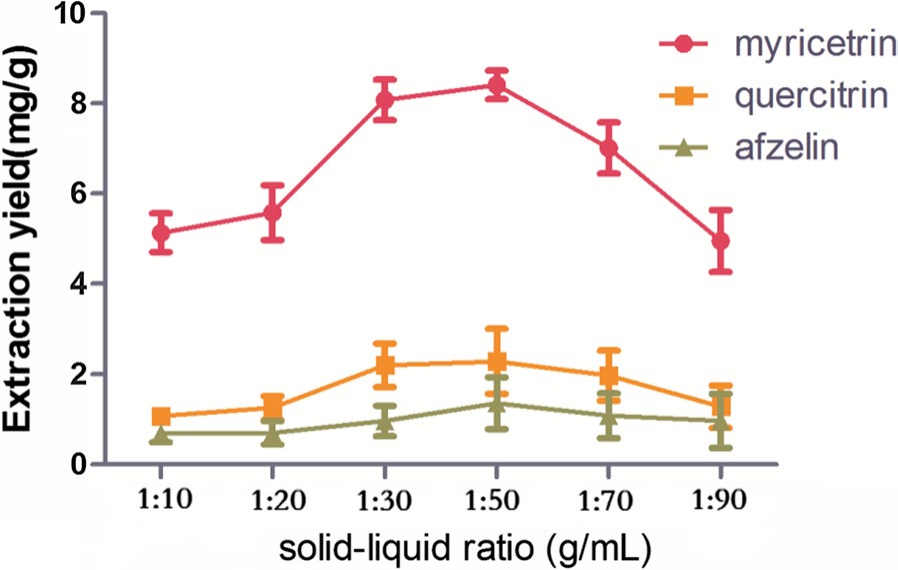
Effect of solid–liquid ratios on extraction yield (n = 3)

On the basis of the above optimized conditions, the effects of solid–liquid ratios on the extraction yields of three target extract were investigated. Each experiment was paralleled three times. In Fig. 7, when the solid–liquid ratio was 1:50, the extraction yield reached maximum. When the ratio of solid–liquid continued to increase, the extraction yield tended to decline. The dissolution rates of myricetrin, quercitrin and afzelin reached the maximum values at the solid–liquid ratio of 1:50. It may be due to the physical properties of the ionic liquids. Therefore, the ratio of 1:50 was chosen for the ratio of solid–liquid.

### Selection of centrifugal speed

**Fig. 8:**
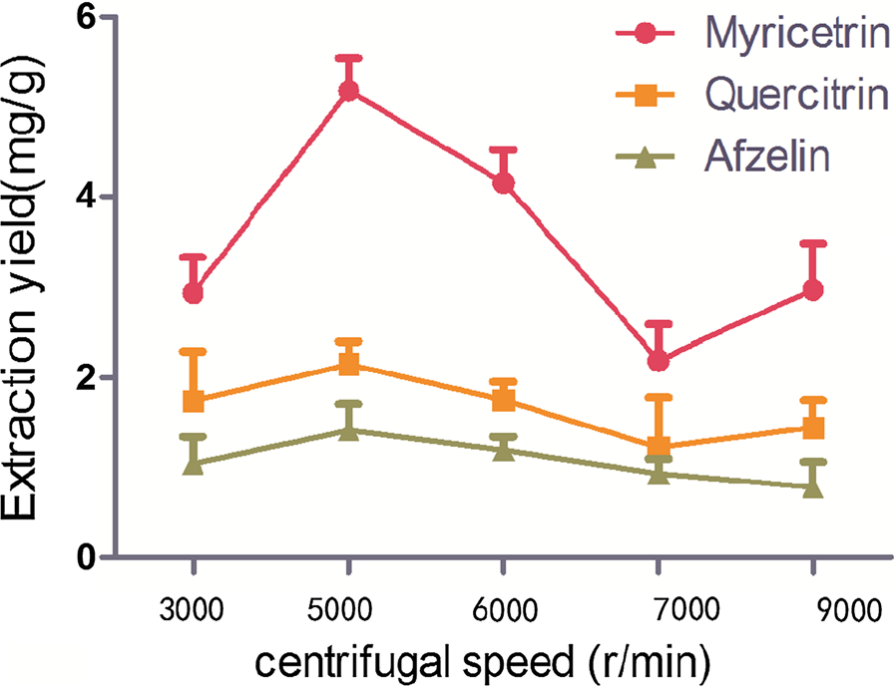
Effect of centrifugal speeds on extraction yield (n = 3)

Under the optimal conditions, five different centrifugal speeds (3000, 5000, 6000, 7000 and 9000 r min^−1^) were chosen to evaluate the effect of centrifugal speed on the extraction yield. The results were shown in Fig. 8, which indicated that the extraction rate reached the maximum at 5000 r min^−1^. Each experiment was paralleled three times. Thus, the centrifugal speed of 5000 r min^−1^ was chosen as the centrifugal speed.

### Optimization the extraction of flavonoids such as myricetrin in *C. chinensi*s leaves

To the best of our knowledge, various parameters play an important role in the optimization of the experimental conditions for the development of a solvent extraction method. The investigated levels of each factor were selected according to the above experiment results of the single-factor. Independent variables with three variation levels are listed in Table 1.

Through the SPSS 19.0, the blank column design orthogonal test was added and the optimum extraction conditions of leaf flavonoids from *C. chinensis* were tested with the comprehensive score as the index. Comprehensive scoring method is based on the importance of each index, the weight of the corresponding indicators is determined, and then the comprehensive scoring method for each group of experiments, the formula (2) was determined as follows. In combination with the activity test of the three compounds in this research group, the three indexes were comprehensively evaluated. Therefore, the weight coefficients of the 3 indexes were 0.5, 0.3 and 0.2, respectively.

2 $$ Test\;score = \sum\nolimits_{i} {(Wi \times Thei - theindex)}. $$

In the present study, all the selected factors were examined by SPSS 19.0 test design. The total evaluation index was used to analyze with statistical method. The analysis results of orthogonal test, performed by statistical software SPSS 19.0, are presented in Tables 2 and 3.

**Table 2:** Results of extreme analysis

No.	1	2	3	4	5	6	Results (extraction yield)			
	A	B	C	D	E		Myricetrin	Quercitrin	Afzelin	Score
1	2	2	1	2	2	3	3.844	0.714	0.354	36.553
2	1	3	3	2	2	2	3.595	0.620	0.343	33.685
3	3	1	1	2	3	3	8.740	1.589	1.151	91.409
4	3	1	3	2	1	2	2.955	0.542	0.292	28.486
5	2	1	2	3	3	2	7.268	1.305	0.831	72.518
6	1	2	1	3	3	2	8.290	1.448	0.975	82.713
7	1	1	2	1	2	3	3.708	0.645	0.387	35.689
8	2	1	3	3	2	1	4.126	0.714	0.405	38.999
9	1	2	3	3	1	3	2.692	0.467	0.256	25.259
10	3	3	2	3	1	3	3.461	0.599	0.244	30.246
11	2	3	1	1	1	2	3.305	0.553	0.316	30.711
12	2	2	2	2	1	1	3.362	0.550	0.298	30.428
13	3	2	3	1	3	1	9.135	1.667	1.232	96.381
14	3	3	1	3	2	1	4.174	0.734	0.424	40.007
15	1	1	1	1	1	1	3.796	0.704	0.362	36.387
16	3	2	2	1	2	2	6.368	1.091	0.620	59.860
17	1	3	2	2	3	1	8.542	1.540	0.995	85.765
18	2	3	3	1	3	3	9.890	1.798	1.169	100
K_1_	299.497	303.489	317.779	359.028	181.517					
K_2_	309.209	331.192	314.506	214.917	244.793					
K_3_	346.389	320.413	322.809	289.742	528.785					
R	37.180	27.704	8.304	144.111	347.268

**Table 3:** Variance analysis of factors

Source	Type III sum of squares	df	F	Sig.	Level (mean ± SD)		
Corrected model	7027.363a	10	4.36	0.059	1	2	3
A	225.269	2	0.699	0.540^b^	46.123 ± 4.962	52.515 ± 6.691	42.027 ± 6.691
B	40.316	2	0.125	0.885^b^	45.488 ± 4.962	45.905 ± 6.691	49.272 ± 6.691
C	611.313	2	1.897	0.244^b^	50.059 ± 4.962	37.234 ± 6.691	53.372 ± 6.691
D	59.085	2	0.183	0.838^b^	44.68 ± 4.962	49.371 ± 6.691	46.613 ± 6.691
E	6091.38	2	18.898	0.005^a^	29.021 ± 4.962	35.765 ± 6.691	75.879 ± 6.691
Error	805.816	5					
Total	36219.509	16					
Corrected total	7833.179	15					

^a^Significant at *p* < 0.05

^b^Significant

*df* degree of freedom

### The results of the intuitionistic analysis

The results of the intuitionistic analysis are shown in Table 2, which results showed that 5 factors (particle size, solid–liquid ratio, ILs concentration, centrifugal speed and ultrasonic time) had great influences on the experimental results. Among them, we could find that particle size was the most important parameter. The factors influencing the extraction yield of leaf flavonoids of *C. chinensis* were listed in a decreasing order as follows: E > D > A > B > C according to their *R* values.

But the estimate of error cannot be calculated by intuitionistic nanalysis which can not accurately reflect the experimental error or a substantial change between the levels [23]. Therefore, in order to be fully and more accurately express the experimental results, further analysis is needed.

### The results of the variance analysis

With the comprehensive score as the index, the variance analysis was carried out by SPSS 19.0 software. In Table 3, the results showed that the E factor (particle size) was extremely significant, and the difference in D factor (centrifugal speed) was also significant. The order and the influence of 5 factors was as follows: E > D > A > B > C. The result was consistent with the visual analysis.

The results were shown in Table 3. A_3_B_2_C_3_D_1_E_3_ was identified as the extraction process as follows: the optimal IL concentration, 0.7 mol/L; ultrasonic extraction time, 50 min; solid–liquid ratio, 1:50; rotational speed, 3000 r min^−1^; and crushing mesh number, 90.

### Comparison between IL-UAE Approach and the Traditional Methods

**Fig. 9:**
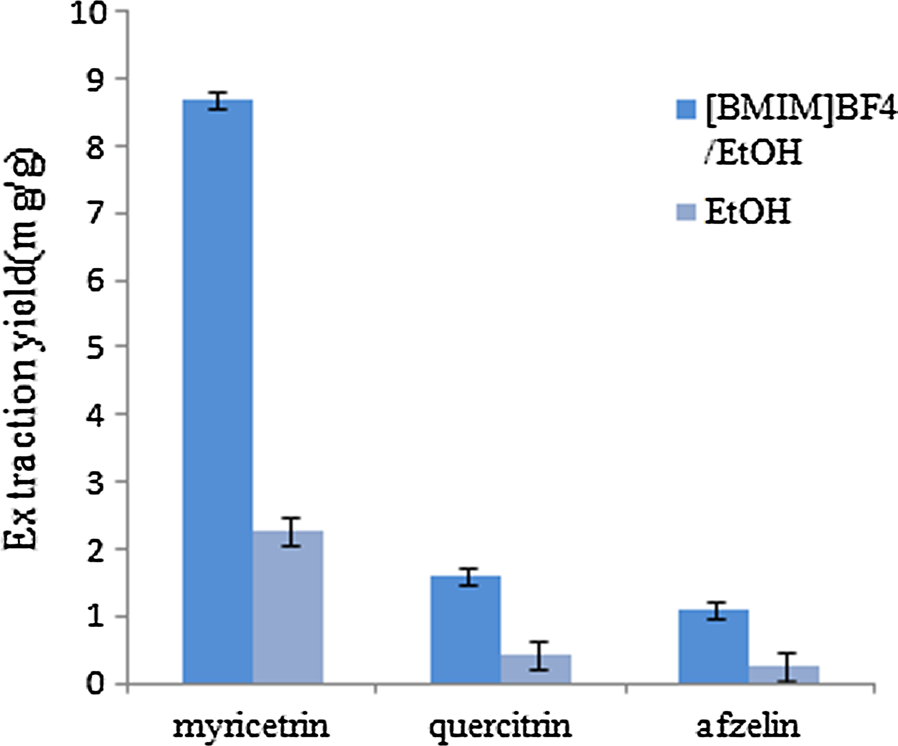
Comparison in extraction yield between the proposed IL-UAE and conventional solvent (n = 3)

In Fig. 9, under the optimal conditions by BMIM BF_4_/70% ethanol extraction, the average contents of myricetrin, quercitrin and afzelin in leaves of *C. chinensi*s were 8.6915, 1.5865 and 1.0920 (mg/g) (*n* = 3) respectively, while the average contents of myricetrin, quercitrin and afzelin in leaves of *C. chinensi*s obtained by traditional solvent-EtOH extracting were 2.2603, 0.4398 and 0.2357 (mg/g) (*n* = 3), respectively. The results showed that the extraction process was optimized by orthogonal test.

### Method validation

#### Determination of sample

Under the optimal conditions, the powder of *C. chinensis* was passed through 90-mesh sieve, and extracted with 1 mL of 0.7 M [BMIM]BF_4_/EtOH in 1:50 of solid–liquid, after 50 min of ultrasonic-aided extraction, extraction solution was obtained. The concentrations of myricetrin, quercitrin and afzelin in sample solution were measured to be 8.6915, 1.5865 and 1.0920 (mg/g), respectively.

#### Repeatability

Six samples of leaves of *C. chinensis* were accurately weighed and the samples were prepared according to the above optimal conditions. The results showed that the relative standard deviation (RSD) of the products were 1.17, 2.96 and 2.00%, indicating the good reproducibility of the experimental method .

The results suggested that myricetrin, quercitrin and afzelin were stable in the ionic liquid solution during the extraction process. Validation studies on these methods indicated that the proposed method was reliable.

#### Precision

The standard sample solution was determined 6 times according to the above chromatographic conditions. The results showed that the precision of the instrument was good with calculated RSDs values of 1.28, 0.72 and 0.43%, respectively, indicating that the precision of the instrument is good and can accurately reflect the amount of the substance.

#### Stability

The sample solutions were prepared under the optimum extraction conditions and placed at room temperature. 10 μL of each solution was injected to chromatographic instrument at 0, 3, 6, 9, 12, and 24 h, respectively. The RSDs of peak areas for myricetrin, quercitrin and afzelin were 2.68, 0.97 and 2.32%. These results indicated that the sample solution was basically stable at room temperature within 24 h.

#### Recovery

Under the optimized conditions detailed above, six samples spiked with myricetrin, quercitrin and afzelin were extracted and the recoveries of myricetrin, quercitrin and afzelin from dried *C. chinensis* leaves were determined to be 100.70, 105.32 and 104.80%, respectively. The RSDs values were 2.90, 2.33 and 2.65%, respectively.

## Conclusions

In this study, an effective method was established to extract myricetrin, quercitrin and afzelin from leaves of *C. chinensis*. Referring to the literature [24–27], it was found that the effect of ILs on extraction of flavonoids, phenols, saponins and terpenoids was better than that of traditional solvents. Compared with traditional methods, the present approach obtained higher extraction yields of myricetrin, quercitrin and afzelin, which were 3–5 times of those obtained with traditional methods, respectively. The optimum conditions for ILUAE were determined by this study. ILs can be recycled by some methods such as vacuum distillation, membrane filtration, salting out, and liquid–liquid extraction [28]. Considering the unique properties of ILs, the developed methods have a promising prospect in sample preparation of Chinese herbal medicine. Therefore, extraction of flavonoids of myricetrin, quercitrin and afzelin in leaves of *C. chinensis* by ion-liquid-assisted extraction provided a theoretical basis for the development and utilization of leaves of *C. chinensis*.

## Abbreviations

ILUAE: ionic liquid based ultrasonic-assisted extraction
HPLC: high-performance liquid chromatography
IL: ionic liquid
ILs: ionic liquids
[HMIM]PF6: 1-hexyl-3-methylimidazolium hexafluorophosphate
[BMIM]BF4: 1-butyl-3-methyl imidazolium tetrafluoroborate
[BMIM]Br: 1-butyl-3-methyl imidazole bromide
[BMIM]PF6: 1-butyl-3- methylimidazolium hexafluorophosphate
LOD: the limit of detection
LOQ: the limit of quantifcation
EtOH: ethanol
MeOH: methanol
RSD: relative standard deviation
